# Effect of Eating Glutinous Brown Rice Twice a Day for 6 Weeks on Serum 1,5-Anhydroglucitol in Japanese Subjects without Diabetes

**DOI:** 10.1155/2020/8847781

**Published:** 2020-10-14

**Authors:** Taiga Nakayama, Yoshio Nagai, Yuka Yasunaka, Takeo Uraguchi, Yukihisa Wada, Masakatsu Sone, Yasushi Tanaka

**Affiliations:** ^1^Division of Metabolism and Endocrinology, Department of Internal Medicine, St. Marianna University School of Medicine, Kawasaki, Kanagawa, Japan; ^2^Department of Internal Medicine, Sakawa Municipal Kohoku Hospital, Takaoka-Gun, Kochi, Japan

## Abstract

We have previously demonstrated that eating glutinous brown rice (GBR) for 1 day or 8 weeks was well accepted and improved glycemic control in patients with type 2 diabetes. The present study evaluated whether eating GBR could also improve glucose metabolism in subjects without diabetes. A prospective 6-week, single-center, randomized, open-label, parallel-group study was carried out in subjects receiving annual medical checkup at our hospital. A total of 42 subjects were randomly assigned to continue their regular diet (RD group) or to switch GBR twice a day (GBR group). The primary outcome was the change in the serum concentration of 1,5-anhydroglucitol (1,5-AG) from baseline after the 6-week dietary intervention. One subject was excluded from the analysis because of a traffic accident. After 6 weeks, the serum 1,5-AG was significantly increased in the GBR group and the mean treatment difference (GBR group − RD group) was 1.1 *µ*g/mL (95% CI: 0.6 to 1.6, *p*=0.022). Body mass index decreased significantly in both groups, with no significant difference between them (*p*=0.210). There were no changes in fasting plasma glucose, fasting insulin, or eating behavior. Intake of GBR for 6 weeks significantly increased serum 1,5-AG in Japanese subjects without diabetes. The increase of 1,5-AG may have been due to the alleviation of postprandial hyperglycemia, which could be effective for the primary prevention of diabetes.

## 1. Introduction

People in Asian countries traditionally consume white rice as a staple food, and it provides more than 30% of the daily energy intake [1]. High intake of white rice was reported to be associated with an increased risk of type 2 diabetes and this relation is stronger for Asians than for Westerners [2, 3].

Lifestyle modification can prevent the onset of type 2 diabetes [4–7]. In particular, dietary modification such as eating vegetables first [8], increasing dietary fiber [9], or incorporating low glycemic index carbohydrates into the daily diet is important and can be more effective than antidiabetic drugs [10–12]. Intake of dietary fiber or whole grain was reported to be associated with a lower risk of developing diabetes [13, 14]. In addition, it was reported the risk of type 2 diabetes is reduced by 16% through replacing 50 g/day of white rice with brown rice [3], and it has been shown that brown rice improves glucose metabolism even in subjects without diabetes [15].

However, it may be difficult for people to add brown rice to the daily diet due to its taste and texture. Rice can be classified as glutinous or nonglutinous. Glutinous rice is very sticky and is mainly used to make rice cakes in Japan. Since glutinous rice is widely accepted by Japanese people as having a good taste and texture, we considered that glutinous brown rice (GBR) might be preferred to brown rice and could be acceptable in the daily diet. We previously demonstrated that eating GBR for 1 day or for 8 weeks improved glycemic control compared with eating white rice, and we showed that GBR also overcame the problem of poor palatability of brown rice [16, 17]. However, these two previous studies were conducted in patients with type 2 diabetes, so the effect of GBR in healthy people without diabetes is unknown.

1,5-Anhydroglucitol (1,5-AG) has a similar structure to glucose which is actively reabsorbed by the renal proximal tubules [18]. Reabsorption of 1,5-AG is competitively inhibited by glucose in the urine, so the serum 1,5-AG concentration is lower in patients with diabetes and several studies have identified 1,5-AG as a useful biomarker of postprandial hyperglycemia [19–21].

Based on the above, the present study was performed to evaluate whether intake of GBR improved glucose metabolism in subjects without diabetes by comparing serum 1,5-AG concentration between subjects eating GBR or a regular diet (RD).

GBR might potentially be effective for primary prevention of diabetes if the increase of serum 1,5-AG is confirmed.

## 2. Materials and Methods

This 6-week, single-center, stratified (24 to 60 years of age (RD group) and 26 to 80 years of age (GBR group), with balanced randomization (1 : 1)), open-label, parallel-group study was designed to evaluate the effect of GBR on parameters of glucose metabolism by comparing with RD in Japanese subjects.

Between August 2017 and September 2017, participants were recruited at the outpatient medical center of our hospital. The inclusion criteria were as follows: (i) persons receiving an annual medical checkup at our hospital, (ii) age of 20 years or older, (iii) fasting plasma glucose (FPG) < 126 mg/dL, (iv) haemoglobinA1c (HbA1c) < 6.5%, (v) body mass index (BMI) ≥ 19 kg/m^2^ and ≤ 27 kg/m^2^, and (vi) persons consuming white rice or white bread in their regular diet. The exclusion criteria were as follows: (i) diabetes mellitus, (ii) dementia, (iii) use of lipid-lowering drugs, (iv) women who were pregnant, possibly pregnant, planned to become pregnant, or were breastfeeding, (v) patients on dialysis, and (vi) persons who were considered to be ineligible for the study for other reasons. Written informed consent was obtained from all participants. This study was performed in accordance with the Declaration of Helsinki and was approved by the ethics committee of our university hospital (no. 3654). In addition, this study was registered with the University Hospital Medical Network Clinical Trials Registry (clinical trial registration number, UMIN000028028).

Participants were randomly assigned to continue their regular diet (RD group) or switch to eating GBR twice a day (GBR group). A computer-generated list of random numbers was used for allocation of the subjects, following simple randomization procedures (computerized random numbers) to 1 of 2 treatment groups. The management of allocations was done by third party unrelated to the researcher enrolling and assessing participants. Also, the allocation sequence was concealed from the researcher enrolling and assessing participants in sequentially numbered, opaque, sealed, and stapled envelopes, and the envelopes were opened only after the enrolled participants completed all baseline assessments and it was time to allocate the intervention. Details of the allocated group were given on the cards contained in sequentially numbered, opaque, sealed envelopes. These were prepared and kept in an agreed location on the secretariat of our hospital (administrator: the executive director). The outcome assessors and the subjects were aware of the allocated arm.

GBR was purchased from Nichirei Foods Inc. (Tokyo, Japan). In order to maintain stable intake of rice, a single trained nutritionist interviewed each participant to assess their daily diet, and the subjects were instructed to eat a specified amount of rice twice a day within 20 minutes. We set the study food intake of each subject as follows: first, we calculated the energy consumption of the staple intake per meal: (i) the calculation of ideal body weight, (ii) the calculation of basal metabolism, (iii) the confirmation of life strength, and (iv) the calculation of staple food intake energy amount per one meal: (i) × (ii) × (iii) × 0.45 × 1/3 (kcal/meal). We consolidated all subjects with staple food intake energy amount as 45% of estimated energy requirement (EER). Next, we calculated the study food intake of each subject from the energy consumption of the staple intake per meal. The energy of each study food is 191 kcal per 100 g (GBR) and 150 kcal per 100 g (WR) (Table 1). Therefore, each study food intake per meal was calculated as follows: 100 (g) ×  (iv) (kcal/meal) ÷ 191 (kcal) (GBR) and 100 (g) × (iv) (kcal/meal) ÷ 150 (kcal). The average dietary energy intake per meal was 262.3 ± 38.5 kcal in the GBR group and 271.3 ± 42.1 kcal in the RD group. On the other hand, the average of each study food intake per meal was 148.2 ± 21.8 g/meal in the GBR group and 161.4 ± 25.1 g/meal in the RD group.

Fasting blood samples were collected before and after the dietary intervention. The primary endpoint of this study was the change of serum 1,5-AG from baseline in each group after the 6-week dietary intervention period. Secondary endpoints were the changes in BMI, FPG, immunoreactive insulin (IRI), and serum C-peptide (s-CPR). We did not change the outcome until the end of this study.

Sakata's Eating Behavior Questionnaire was completed to evaluate changes in eating behavior in the two groups before and after the dietary intervention. This questionnaire has 55 questions, of which we used 52 questions classified into 7 categories: (i) understanding of body constitution and weight, (ii) motivation for eating, (iii) substitution eating and drinking, (iv) feeling of satiety, (v) eating style, (vi) contents of meals, and (vii) abnormalities of eating rhythm [22, 23]. The scores for each question were determined on a 4-point Likert scale (1 = “never”; 2 = “sometimes”; 3 = “often”; 4 = “always”). Each category was evaluated by calculating a summed score, with a lower score indicating better eating behavior. The total Sakata score was also calculated by adding the scores for each category.

Because this is a pilot study, the sample size was based on practical considerations rather than a statistical estimate. It was expected that 42 subjects could be enrolled during the registration period. The Shapiro–Wilk normality test was used to assess whether or not variables had a normal distribution, while the *F*-test was employed to investigate the homogeneity of variance. Continuous variables were expressed as the mean ± SD or SEM. The paired *t*-test was used to compare within-group changes between before and after the dietary intervention, while Student's *t*-test was used to compare characteristics between the groups. The impact of the baseline value on subsequent changes was determined by analysis of covariance (ANCOVA) using the baseline measurement as the covariate and intervention group as fixed effect. All analyses were performed with JMP version 13 (SAS Institute Inc., Cary, NC, USA) and Bell Curve for Excel (Social Survey Research Information Co., Ltd, Japan). Differences were considered to be significant if the probability value (*p*) was less than 5%. In addition, the additional analysis such as subgroup analysis was not conducted in this study.

We did not change all the above methods until the end of this study.

## 3. Results

**Figure 1:**
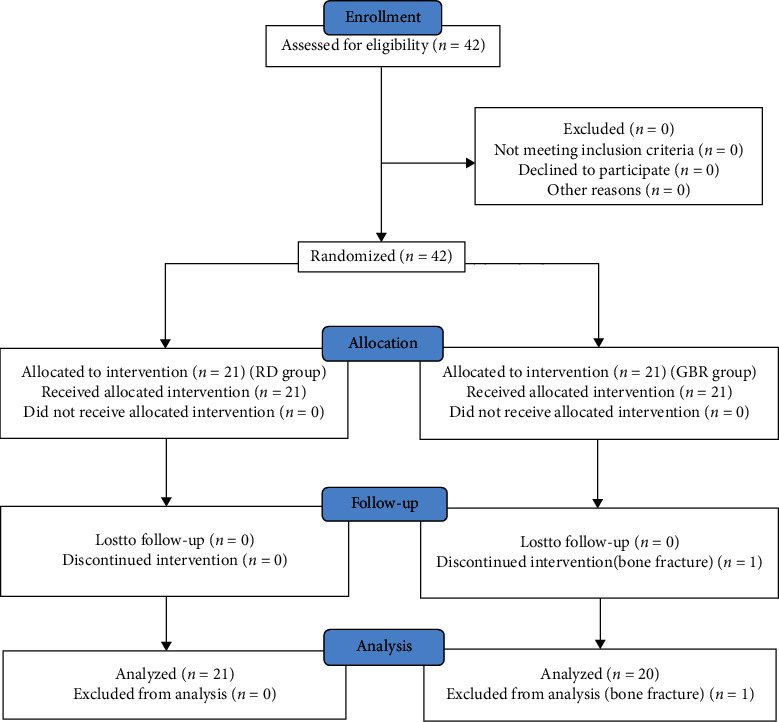
Flow diagram showing the disposition of the subjects.

A total of 42 subjects were enrolled in this study. None of them had previously been eating GBR on a daily basis. One subject was excluded from analysis because of emergency hospitalization to treat a fracture caused by a traffic accident. The other 41 subjects (7 men and 34 women) completed the study and were defined as the per-protocol set for analyses (Figure 1). One of the GBR groups was withdrawn due to bone fracture. Subjects visited our hospital at the time of randomization (baseline) and at 6 weeks (endpoint) between August 2017 and September 2017.

All the subjects were Japanese and 34 (82.9%) were women. The mean age was 44.3 ± 12.0 years, the mean BMI was 21.6 ± 1.7 kg/m^2^, the mean FPG was 94.7 ± 7.7 mg/dL, and the mean HbA1c was 5.2 ± 0.3%. Baseline characteristics of the RD group and the GBR group are shown in Table 2. There were no significant differences between the two groups with regard to gender, age, BMI, serum 1,5-AG, FPG, HbA1c, the average energy intake from staple foods per meal, and the average of each study food intake per meal.

**Figure 2:**
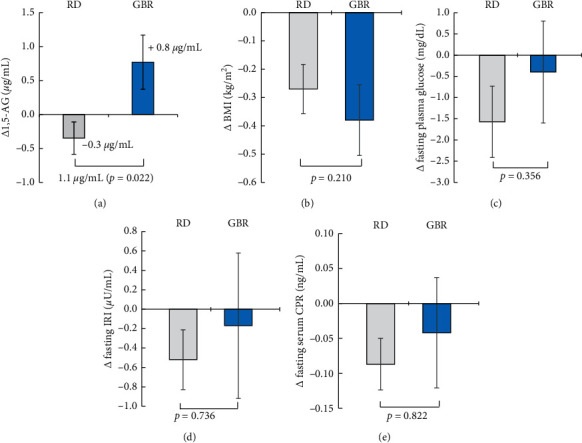
Change of the mean (a) serum 1,5-AG, (b) BMI, (c) FPG, (d) IRI, and (e) s-CPR between baseline and 6 weeks. Data are the mean ± standard error. Analysis of covariance (ANCOVA) including baseline value as a covariate and intervention group as fixed effect. The serum 1,5-AG was significantly increased in the GBR group and the mean treatment difference (GBR group − RD group) was1.1 *µ*g/mL (*p*=0.022).

After 6 weeks, the serum 1,5-AG concentration increased from 20.6 ± 1.4 to 21.4 ± 1.4 *µ*g/mL (*p*=0.072) in the GBR group, while it decreased slightly from 21.3 ± 1.9 to 20.9 ± 1.8 *µ*g/mL (*p*=0.157) in the RD group. The change of serum 1,5-AG from baseline showed a significant difference between the two groups, and the mean difference (GBR group − RD group) was 1.1 *µ*g/mL (95% CI: 0.6 to 1.6, *p*=0.022; Figure 2(a)).

BMI showed a significant decrease in both groups from 21.7 ± 0.5 to 21.4 ± 0.4 kg/m^2^ in the RD group (*p*=0.005 vs. baseline) and from 21.5 ± 0.2 to 21.1 ± 0.2 kg/m^2^ in the GBR group (*p*=0.007 vs. baseline). There was no significant difference in the change in BMI between the two groups (*p*=0.210; Figure 2(b)). Fasting concentration of plasma glucose (*p*=0.356; Figure 2(c)), IRI (*p*=0.736; Figure 2(d)), and s-CPR (*p*=0.822; Figure 2(e)) were unchanged in both groups.

**Figure 3:**
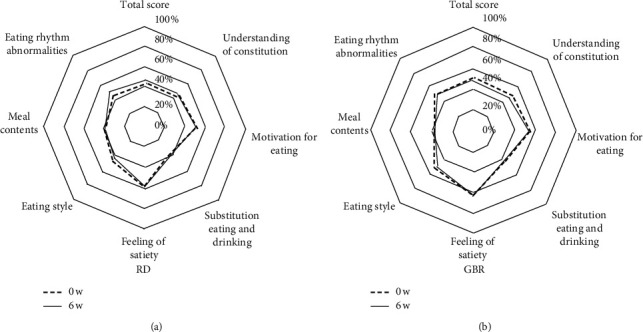
Changes of the scores for Sakata's Eating Behavior Questionnaire in (a) the RD group and (b) the GBR group. Data show the percentage (%) of the maximum scores in each category. The maximum scores for each category were as follows: (i) 28 points, (ii) 40 points, (iii) 16 points, (iv) 16 points, (v) 20 points, (vi) 36 points, (vii) 32 points, and total score: 188 points. RD, regular diet; GBR, glutinous brown rice.

The total Sakata score decreased from 95.3 ± 3.8 to 91.0 ± 5.2 points in the GBR group (*p*=0.301), whereas it increased slightly from 86.3 ± 3.1 to 86.6 ± 3.3 points in the RD group (*p*=0.905 = ). The change from baseline did not show a significant difference between the two groups (*p*=0.547). There were also no significant changes in each Sakata score category in both groups after 6 weeks (Figure 3).

None of the subjects dropped out of the study because of problems with the taste of GBR, and there were no adverse effects in either group during the 6-week study period. In addition, subgroup analysis was not conducted in this study.

## 4. Discussion

This study demonstrated that healthy subjects without diabetes found the intake of GBR to be acceptable. The serum 1,5-AG concentration increased significantly in the GBR group after eating GBR twice a day for 6 weeks, but there were no concomitant changes in body weight, FPG, IRI, s-CPR, or eating behavior when compared with the control group. These results suggest that the increase of serum 1,5-AG was not due to weight loss, reduction of FPG, increased endogenous insulin secretion, or improvement of eating behavior due to intake of GBR. Accordingly, GBR may have an indirect effect on serum 1,5-AG.

Several studies have identified the serum 1,5-AG level as a useful biomarker for postprandial hyperglycemia [18–20]. The elevation of serum 1,5-AG in the present study was consistent with our previous finding that eating GBR improved postprandial glucose (evaluated by continuous glucose monitoring) in patients with T2DM [16]. The mechanism by which intake of GBR affects the serum 1,5-AG concentration is uncertain, and whole grain foods contain various nutrients that include dietary fiber, vitamins, and minerals. GBR contains far more dietary fiber than white rice (3.2 g vs. 0.2 g per 100 g; Table 1). It was reported that dietary fiber in whole grain foods could significantly reduce the postprandial plasma glucose concentration [24], and foods derived from whole grains are classified as having a low glycemic index. GBR also contains far more *γ*-oryzanol than white rice (27.4 mg vs. 0 mg per 100 g). Endoplasmic reticulum (ER) stress is profoundly involved in the dysfunction of pancreatic *β*-cells under hyperglycemia. *γ*-Oryzanol has shown to reduce ER stress in pancreatic *β*-cells [25]. In the present study, increased intake of dietary fiber and/or *γ*-oryzanol in the GBR group might have reduced postprandial blood glucose excursions, leading to elevation of the serum 1,5-AG.

With regard to eating behavior, subjects allocated to the GBR group were required to eat GBR twice a day for 6 weeks. There were no differences in their eating behavior between before and after the 6-week intervention period, apart from the intake of GBR, suggesting that there was no bias of eating behavior due to the use of GBR as staple food.

In the present study, the change of serum 1,5-AG was small. However, it would be of note that only 1,5-AG showed a significant difference, even though other parameters such as body weight, FPG, IRI, and eating behavior did not change. In healthy subjects, it has been reported that an increase of serum 1,5-AG by 1.0 *µ*g/mL was equivalent to a decrease of postprandial plasma glucose by 5 mg/dL [26]. This result suggests that eating GBR regularly might prevent developing diabetes.

The present study had several limitations. First, we only performed laboratory tests in the fasting state, so we did not obtain data about postprandial plasma glucose. Further investigation will be needed to confirm the effect of GBR on postprandial plasma glucose in subjects without diabetes. Second, we did not assess urinary glucose excretion. We could not exclude the subject who has renal glycosuria or mild glucose intolerance which are known to influence serum 1,5-AG concentration.

Despite these potential sources of bias, our findings revealed that intake of GBR led to a significant increase in the serum 1,5-AG concentration compared with the RD in Japanese subjects. The increase of serum 1,5-AG might be due to the improvement of postprandial hyperglycemia, which indicated that GBR might potentially be effective for primary prevention of diabetes.

## 5. Conclusions

Continuous intake of GBR for 6 weeks increased the serum 1,5-AG concentration in Japanese subjects without diabetes compared with RD. There were no significant differences between the two groups with regard to BMI, FPG, fasting serum CPR, and fasting IRI. Therefore, it was suggested that the improvement of the serum1,5-AG concentration in GBR compared with RD may be contributed to the improvement of postprandial blood glucose level.

## Figures and Tables

**Table 1:** Ingredient of the study food.

	RD	GBR
Total energy (kcal)	150	191
Carbohydrate (g)	33.0	38.5
Protein (g)	2.0	3.9
Fat (g)	0.4	2.3
Dietary fiber (g)	0.2	3.2
*γ*-Oryzanol (mg)	0.0	27.4

**Table 2:** Characteristics of the participants.

	RD (*n* = 21)	GBR (*n* = 20)	*p* value
Men/women	4/17	3/17	0.738
Age (years)	41.9 ± 10.1	46.8 ± 13.4	0.190
BMI (kg/m^2^)	21.7 ± 2.1	21.5 ± 1.1	0.657
1,5-AG (*μ*g/mL)	21.3 ± 8.9	20.6 ± 6.4	0.792
FPG (mg/dL)	94.5 ± 7.7	94.9 ± 10.1	0.878
HbA1c (%)	5.2 ± 0.2	5.1 ± 0.3	0.335
GA (%)	14.2 ± 1.2	14.5 ± 1.1	0.409
IRI (*µ*U/mL)	5.1 ± 2.2	4.9 ± 2.4	0.782
s-CPR (ng/mL)	1.2 ± 0.4	1.1 ± 0.4	0.544
LDL-C (mg/dL)	113.8 ± 26.4	112.2 ± 19.5	0.835
HDL-C (mg/dL)	64.2 ± 12.4	67.1 ± 14.1	0.498
TG (mg/dL)	83.8 ± 43.2	68.3 ± 33.2	0.215
Energy (kcal/meal)	271.3 ± 42.1	262.3 ± 38.5	0.485

Data are expressed as the mean ± SD for continuous variables or the number for categorical variables. BMI, body mass index; 1,5-AG, 1,5-anhydroglucitol; FPG, fasting plasma glucose; HbA1c, hemoglobin A1c; GA, glycoalbumin; IRI, immunoreactive insulin; s-CPR, serum C-peptide; TG, triglycerides; energy, energy from staple food per meal.

## Data Availability

The data used to support the findings of this study are available from the corresponding author upon request.
